# Sounding out the dynamics: a concise review of high-speed photoacoustic microscopy

**DOI:** 10.1117/1.JBO.29.S1.S11521

**Published:** 2024-02-05

**Authors:** Soon-Woo Cho, Van Tu Nguyen, Anthony DiSpirito, Joseph Yang, Chang-Seok Kim, Junjie Yao

**Affiliations:** a Duke University, Department of Biomedical Engineering, Durham, North Carolina, United States; b Pusan National University, Engineering Research Center for Color-Modulated Extra-Sensory Perception Technology, Busan, Republic of Korea

**Keywords:** photoacoustic microscopy, optoacoustic microscopy, high-speed light source, high-speed scanning, optical acoustic detection, computational techniques, deep-learning

## Abstract

**Significance:**

Photoacoustic microscopy (PAM) offers advantages in high-resolution and high-contrast imaging of biomedical chromophores. The speed of imaging is critical for leveraging these benefits in both preclinical and clinical settings. Ongoing technological innovations have substantially boosted PAM’s imaging speed, enabling real-time monitoring of dynamic biological processes.

**Aim:**

This concise review synthesizes historical context and current advancements in high-speed PAM, with an emphasis on developments enabled by ultrafast lasers, scanning mechanisms, and advanced imaging processing methods.

**Approach:**

We examine cutting-edge innovations across multiple facets of PAM, including light sources, scanning and detection systems, and computational techniques and explore their representative applications in biomedical research.

**Results:**

This work delineates the challenges that persist in achieving optimal high-speed PAM performance and forecasts its prospective impact on biomedical imaging.

**Conclusions:**

Recognizing the current limitations, breaking through the drawbacks, and adopting the optimal combination of each technology will lead to the realization of ultimate high-speed PAM for both fundamental research and clinical translation.

## 1 Introduction

**Fig. 1:**
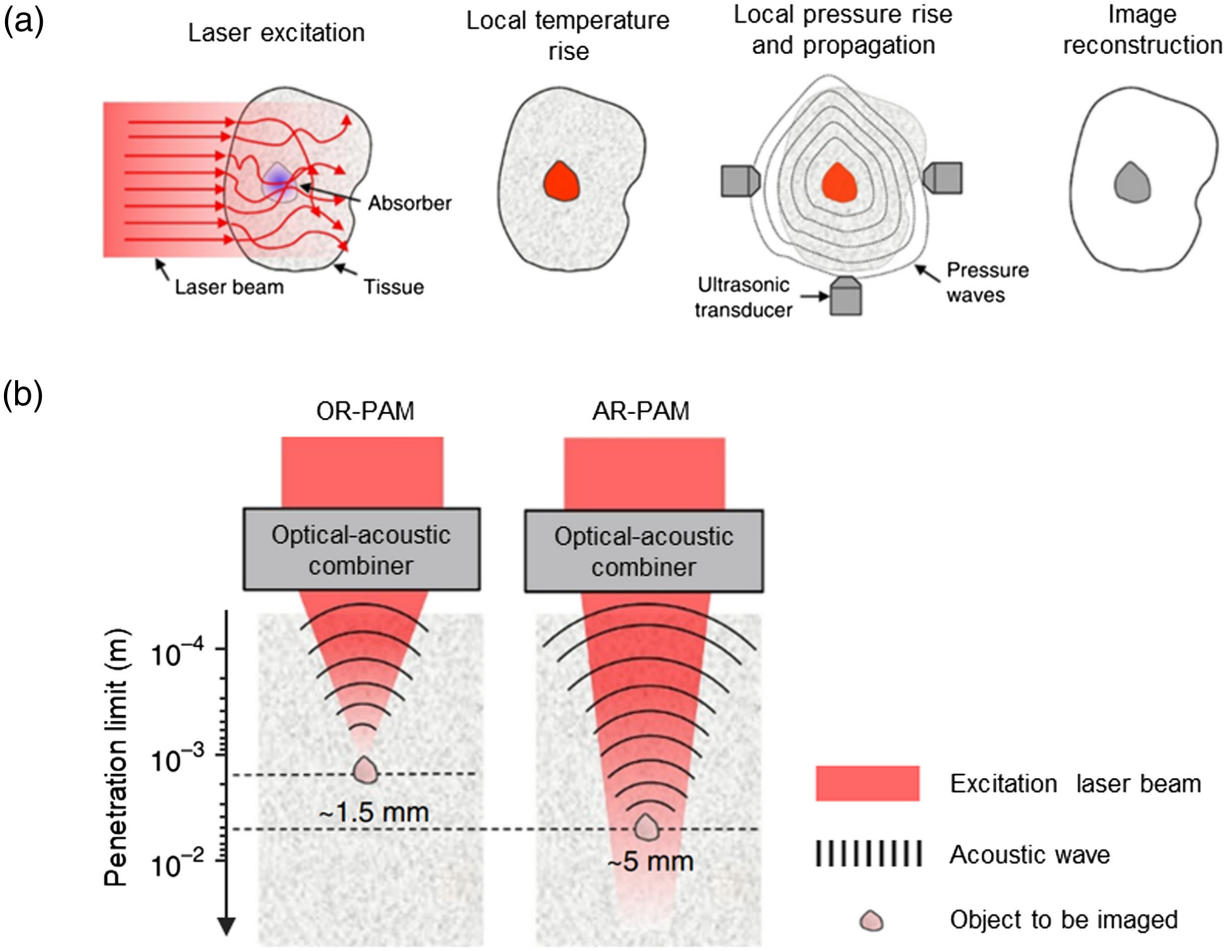
Principle of PA imaging and penetration limits of PAM. (a) Illustration of PA imaging principle. (b) Penetration depth of OR-PAM and AR-PAM with different optical and acoustic focusing configurations. The images are reprinted with permission from Ref. 4.

Photoacoustic (PA) imaging (PAI) has emerged as a promising biomedical technique based on the PA effect, wherein ultrasonic emission occurs upon the absorption of a short-pulsed laser by irradiating objects, such as biological tissues.^1^^–^^3^ The resultant ultrasonic waves, termed PA waves, are captured by a transducer to form PA images for diverse applications [Fig. 1(a)]. PA microscopy (PAM) is a prevalent form of PA imaging that achieves micron-level spatial resolution by focusing either the optical or acoustic beam or both. According to the focusing type, the PAM system is categorized into optical-resolution PAM (OR-PAM) and acoustic-resolution PAM (AR-PAM).^5^^,^^6^ Using wide-field optical excitation, AR-PAM can achieve a 3 to 5 times larger penetration depth than OR-PAM [Fig. 1(b)]. By contrast, because an optical focus is approximately 10 times tighter than an acoustic focus, OR-PAM excels in delivering higher resolution, which has the capability to image physiological and pathological changes in single capillaries.^7^ Furthermore, the remarkable optical absorption contrast makes PAM a promising imaging tool across various research domains including biology, dermatology, neurology, oncology, ophthalmology, and pathology.^8^

Traditional PAM systems employ a confocal and coaxial configuration for the excitation light and acoustic emission beams, maximizing the signal-to-noise ratio (SNR) and spatial resolution. Because the acquisition of three-dimensional (3D) images is conducted by raster scanning of the PA probe over the sample, with the typical lasers and scanning systems, the combination of a low pulse repetition rate (PRR) and a fine scanning step size has led to a prolonged imaging time. Consequently, typical OR-PAM faces limitations in capturing dynamic tissue information, such as transient drug responses in brain microvasculature.^9^ Thus, many efforts have concentrated on improving the imaging speed of PAM to monitor the rapid dynamic changes in biomedical research.

Fundamentally, the imaging speed of PAM is limited by the PRR of the light source because a single laser pulse can generate a time-resolved PA signal. Assuming that there is no need for signal averaging for improving the SNR, the laser PRR generally corresponds to the A-line rate of PAM. Various types of pulsed lasers can be classified according to the wavelength, pulse duration, pulse energy, and PRR. Among them, Q-switched lasers, which usually have a nanosecond pulse duration, have been mainly used in PAI studies.^10^ Because the first-generation PAI needed the millijoule (mJ) level of pulse energy for the wide-field illumination, the initial research of PAM was conducted with slow and bulky lasers such as an optical parametric oscillator (OPO),^11^ a Ti:Sapphire laser,^12^ or a dye laser^13^ pumped by a flash-pumped solid-state laser (FPSS). However, as the need for speed improvement increases in the high-resolution scheme, greater attention was paid to the high-PRR lasers, which can replace conventional lasers.^14^ Therefore, in recent years, fiber lasers have been increasingly used to achieve ultra high-speed PAM, with a short pulse duration (<10  ns), a large pulse energy (>hundreds nJ), and an ultra-high PRR (hundreds kHz∼MHz).^15^^–^^24^

From the perspective of a scanning system, a wide field-of-view (FOV) with a short imaging time is another need for high-speed PAM. Because diverse temporal and spatial scales exist in biological functions, a strategy to match the FOVs and scanning speed is necessary. Because typical PAM is sensitive to significant motion artifacts, such as those induced by respiratory movements, high-speed scanning methods with large FOVs have been reported to maximize the functional imaging capabilities of PAM. Initially, researchers have explored the use of faster scanning stages, such as voice coil scanners^25^ and slider-crank scanners,^26^^,^^27^ while maintaining the same setup as conventional PAM systems. These mechanical scanning approaches allowed the systems to achieve a high SNR; however, they consistently faced speed limitations due to the bulky scanners and associated vibrations. Moreover, scanning speeds were still insufficient to meet the desired requirements for dynamic imaging. On the other hand, the optical scanning approach employs stationary PA signal detection without any mechanical scanning of the sample/transducer; thus, the scanning speed is only limited by the laser’s pulse rate or the speed of PA signal generation.^28^ The use of a water-immersible mirror driven by a microelectromechanical systems (MEMS) scanner^29^^–^^31^ introduced an innovative approach that led to a significant scanning speed improvement in PAM. Following this breakthrough, other optical scanning solutions such as galvanometer scanners^32^ and polygon scanners^22^ have emerged; each offers a different configuration to optimize the scanning speed. These advancements have pushed the boundaries of PAM speed, approaching the physical limit dictated by the speed of sound.

To further improve the imaging speed of PAM, in addition to the scanning mechanism, it is necessary to explore alternative approaches that can expedite the detection of PA signals. Optical sensors have emerged as a promising avenue, with a focus on achieving faster signal detection speeds. These optical sensors offer several advantages, including their compact size, wide reception angle, broad detection bandwidth, strong responsiveness to low-frequency signals, and seamless integration with the PA light path. Notably, the sensitivity of optical sensors tends to exhibit less dependence on sensor size, resulting in superior sensitivity compared with piezoelectric transducers of similar dimensions, particularly at higher frequencies (>2.5  MHz).^33^

Computational techniques to improve the imaging speed of PAM are inextricably linked to hardware limitations, such as the PRR of laser sources and scanning mechanisms. These software techniques serve to either circumvent or correct some of the hardware limitations of high-speed PAM systems. When scanning speed limitations are reached from the hardware perspective, due to the limited PRR of laser sources or scanning mechanisms, undersampling is often performed by covering the same FOV with fewer sampling events.^34^ When using traditional pulsed laser sources, this undersampling typically results in a lower sampling density and violates the Nyquist sampling theorem, causing overall poorer image quality and introducing aliasing (spatial undersampling).^34^^,^^35^ When performing localization microscopy, undersampling typically takes the form of using fewer frames, thus resulting in images with a poorer SNR or incomplete structures due to insufficient sampling (temporal framewise undersampling).^36^ Therefore, many computational techniques for high-speed PAM aim to overcome the tradeoff between imaging speed and its subsequent degradation of image quality through a process of image restoration. Other computational techniques speed up PAM image formation by attempting to correct imperfections in the scanning mechanism, such as by quickly restoring misaligned/distorted aspects of the scanning path or denoising low-energy images acquired within certain laser dosage limits.^22^^,^^37^^–^^39^

In this concise review, we cover a brief overview of (i) light sources, (ii) scanning and detection, and (iii) computational techniques to improve the PAM imaging speed. Then we introduce the recent technical advances compared with conventional PAM system in the last 5 years or so. Finally, we conclude by briefly discussing the remaining challenges and future potential for high-speed PAM.

## 2 Light Sources

The pulsed light source is one the most critical components of PAM, which often determines its maximal A-line rate, SNR, and lateral spatial sampling density. Accelerating the speed of PAM requires the engineering of novel light sources that can provide a high PRR, high pulse energy, and ideally, multi-wavelengths for functional studies, which are usually competing parameters for any given laser type.

### 2.1 Conventional Excitation Lasers for PAM

In traditional PA imaging, FPSS lasers were used due to their extremely high energy (∼ several J), which enables obtaining the various scales of PA image and pumping the dye laser or OPO.^11^^–^^13^ However, the FPSS lasers has a low PRR (10∼100  Hz) and a limited lamp lifetime of a few million pulses.^40^^,^^41^ Thus, diode-pumped solid-state (DPSS) lasers, utilizing a high-power semiconductor laser diode as a pumping source for Q-switching, have been widely used with a higher speed (∼kHz range).^42^ Laser diodes can optimize crystal efficiency for the lowest possible pump energy by tuning the wavelength controlled by temperature and driving current, which allows DPSS lasers to have high energy efficiency, good beam quality, high compactness, and long lifetime.^43^ PAM can also be implemented using Q-switched microchip lasers, which are alignment-free monolithic solid-state lasers (10  kHz∼100  kHz). The microchip cavity consists of a thick disk sandwiched between mirrors with extremely short cavity lengths, which allows the laser to oscillate on a single longitudinal mode generating a nanosecond Q-switched pulse.^44^ Microchip lasers can reach up to above 100 kHz with passive Q-switching, but in practice, PAM typically operates at tens of kHz PRRs with a few microjoules due to trade-offs between the PRR and pulse energy.^45^ When a gain crystal (e.g., Nd:YAG or $\mathrm{Nd}:{\mathrm{YVO}}_{4}$) is overloaded by high pump power for a high PRR (hundreds of kHz), the thermal lensing effect and thermal damage occur, leading to serious performance degradation.^46^ Thus, solid-state lasers with crystals are not capable of providing high-energy nanosecond pulses at a high PRR.

### 2.2 High-Speed Excitation Lasers

In terms of thermal resistance, fiber lasers can be a solution for high-speed PAM. The optical fiber has the merit of a large surface-to-volume aspect ratio, which prevents the thermal lensing effect by facilitating heat dissipation. This advantage enables the fiber lasers to withstand the frequent on/off intensity modulation and high peak power at a high PRR. Thus, current high-speed PAM systems have mostly adopted fiber lasers to provide a stable output with microjoule energy at the MHz-level PRR.^16^^–^^18^^,^^20^^–^^22^

#### 2.2.1 Single wavelength excitation

Shi et al. first discussed the potential of Q-switched fiber lasers as a high-speed OR-PAM light source in 2010.^14^ Because the optical absorption of hemoglobin at 1064 nm is low, a custom-built passively Q-switched ytterbium doped (Yb-doped) fiber laser at 1075 nm with a PRR of 100 kHz and a pulse energy of a 13  μJ was converted to 537 nm with a pulse energy of 0.06  μJ and a pulsewidth of 250 ns pulse, by coupling the 1075 nm light into a potassium titanium oxide phosphate (KTP) frequency-doubling crystal. The capability of fiber lasers with a high PRR (hundreds of kHz) was demonstrated for real-time OR-PAM, though the long pulse duration resulted in a poor SNR. The same group later demonstrated *in vivo* OR-PAM using a 532-nm commercial Yb-doped fiber laser with a PRR up to 600 kHz.^24^ The short pulse duration (∼1  ns) met the thermal and stress-confinement conditions, and the relatively high pulse energy (∼150  nJ) enabled high-contrast *in vivo* images of microvasculature in a mouse ear. In 2015, Yao et al. adopted a picosecond fiber laser with a PRR of 500 kHz for fast functional PAM of a mouse brain based on the single-wavelength pulse-width method.^15^ Despite using only single-wavelength excitation, the oxygen saturation of hemoglobin (${\mathrm{sO}}_{2}$) in the mouse brain was mapped by acquiring the relative concentration of oxyhemoglobin and deoxyhemoglobin under nanosecond and picosecond excitations, respectively. This fiber laser-based OR-PAM with a submersible MEMS scanner achieved a C-scan rate of 1 Hz over an FOV of $3\times 2\;{\mathrm{mm}}^{2}$. In 2018, Allen et al. demonstrated high-speed laser-scanning OR-PAM using a custom-built fiber laser operating at a PRR of 2 MHz.^16^ The maximum PRR, fundamentally limited by the depth-range ambiguity condition, was set at 2 MHz assuming the absorbers are located at a depth range of 750  μm. In combination with an MHz fiber laser and a fiber-optic ultrasound detector scanned by a galvanometer, an *in vivo* OR-PAM image of the mouse ear with an FOV of $10\times 10\;{\mathrm{mm}}^{2}$ was obtained within 8 s.

#### 2.2.2 Multi-wavelength excitation

The fast advancement of commercial nanosecond fiber lasers has in turn promoted the development of multi-wavelength light sources for high-speed PAM capable of functional imaging. Supercontinuum (SC) sources have been increasingly applied for multi-wavelength excitation in PAM.^47^^–^^52^ The use of highly nonlinear fibers, such as photonic crystal fibers, broadens the output light spectrum to hundreds of nanometers, which leads to a low energy density within each specific band. Recently, Chang et al. reported the use of a commercial SC source with a PRR of up to 1 MHz for multispectral PAM and OCT.^17^ Two wavelength bands around 528 nm and 558 nm light with a 10 nm bandwidth were used to image ${\mathrm{sO}}_{2}$ of the mouse ear. However, the two PA signals, generated at a PRR of 500 kHz, were respectively averaged 250 times to improve the SNR.

**Fig. 2:**
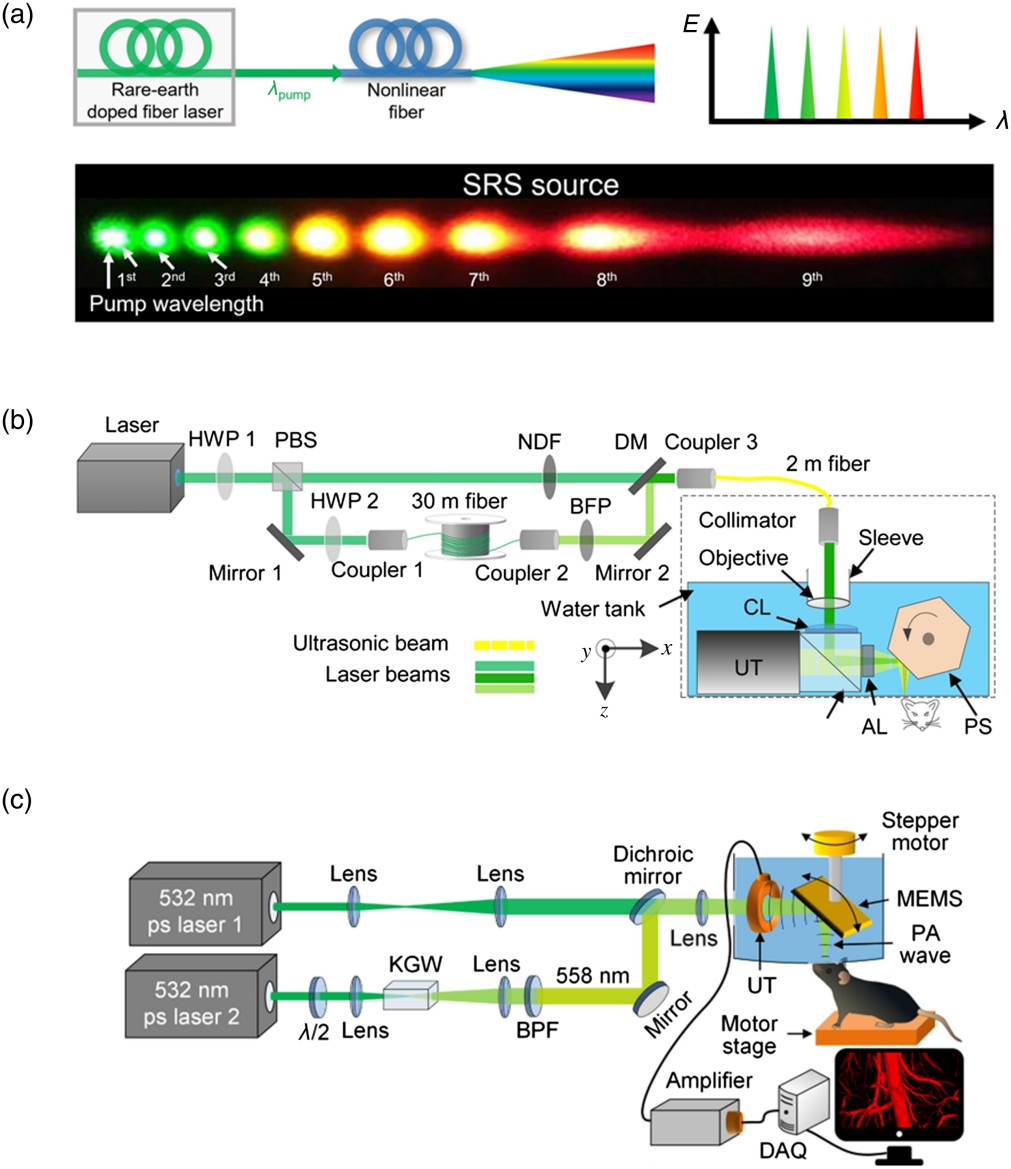
High-speed PAM using an SRS source. (a) Schematic illustration of the SRS effect in an optical fiber and the discrete Raman spectrum.^10^ (b) Schematic of the high-speed PAM system using an SRS source with an optical fiber pumped by a 1-MHz nanosecond pulsed laser.^20^ (c) Schematic of the high-speed PAM using an SRS source with a Raman crystal pumped by a picosecond pulsed laser.^21^ Images (a), (b), and (c) are reprinted with permission from Refs. 10, 20, and 21, respectively.

The stimulated Raman scattering (SRS) source is another technique for multi-wavelength PA excitation; it generates cascaded Stokes outputs based on the nonlinear SRS effect in optical fibers. Because SRS sources have highly condensed energy in a narrow band converted from the pump light [Fig. 2(a)], they have been adopted for implementing high-speed multi-wavelength PAM.^18^^–^^23^ Hajireza et al. first reported an SRS source pumped by a Yb-doped fiber laser for multi-wavelength OR-PAM and conducted *in vivo* functional imaging of the mouse ear.^23^ Although the pump laser output had a PRR of up to 600 kHz, the carbon fibers were imaged at a PRR of 160 kHz, and the *in vivo* experiment was demonstrated at a PRR of 40 kHz, where the desired wavelengths at 545 and 558 nm had the maximum energy through a polarization-maintaining single-mode fiber (PM-SMF). Cho et al. reported the optimal generation of SRS sources with a PRR of 300 kHz for high-speed molecular PAM.^19^ To generate the various wavelengths with sufficient energy, 10 Stokes SRS wavelengths ranging from green to red were optimized with the pulse energy of 116∼756  nJ through a PM-SMF. By employing the optimized wavelengths, *in vivo* molecular PAM was used to distinguish the gold nanoparticles from the blood vessels at a PRR of 300 kHz. Wang’s group employed a 1-MHz dual-wavelength pulse laser system based on the SRS effect for high-speed PAM.^18^^,^^20^ The pump laser was a Q-switched fiber laser with a PRR of 1 MHz. Using a dual-path configuration with optical delay [Fig. 2(b)], a pump wavelength (532 nm) and a Raman Stokes wavelength (558 nm) were generated with a 150 ns interval. This study demonstrated functional OR-PAM at a 1 MHz A-line rate, which allowed for the observation of the rapid ${\mathrm{sO}}_{2}$ change in the mouse ear after intravenous epinephrine injection. He et al. proposed another 1-MHz dual-wavelength PAM using a crystal-based SRS source^21^ and combined a picosecond fiber laser and a potassium gadolinium tungstate $\left[\mathrm{KGd}{\left({\mathrm{WO}}_{4}\right)}_{2}\right]$ crystal with high Raman gain coefficient to enhance the SRS effect. As shown in Fig. 2(c), a dual-wavelength PAM was implemented using two 532-nm picosecond-pulsed lasers, one for the 532-nm path and the other for the first-Stoke 558-nm path. At a PRR of 1-MHz, PAM was used to observe the single-impulse-stimulated initial dip from cerebral micro-vessels without repeated stimulation. In a recent work, Zhu et al. developed high-speed PAM based on an SRS source pumped by a fiber laser with a PRR of up to 2 MHz.^22^ Dual-wavelength excitation at 532 nm and 558 nm with a PRR of 800 kHz enabled functional imaging of rapid whole-brain hemodynamics, with a volumetric frame rate of 2 Hz over an FOV of $11\times 7.5\times 1.5\;{\mathrm{mm}}^{3}$.

## 3 Scanning Systems

High-speed PAM systems have been focused on expanding the FOV while preserving high-sensitivity detection. Although the scanning time is technically determined by the PRR of the laser and scanning mechanism, it is fundamentally constrained by the speed of sound in biological tissues.^16^ Various high-speed scanning methods have been demonstrated for PAM, including mechanical scanning methods, such as voice-coil scanners and slider-crank scanners, and optical scanning methods, such as galvanometer scanners, MEMS scanners, and polygon-mirror scanners. Different scanning methods have their own merits and drawbacks.

### 3.1 Mechanical Scanning

**Fig. 3:**
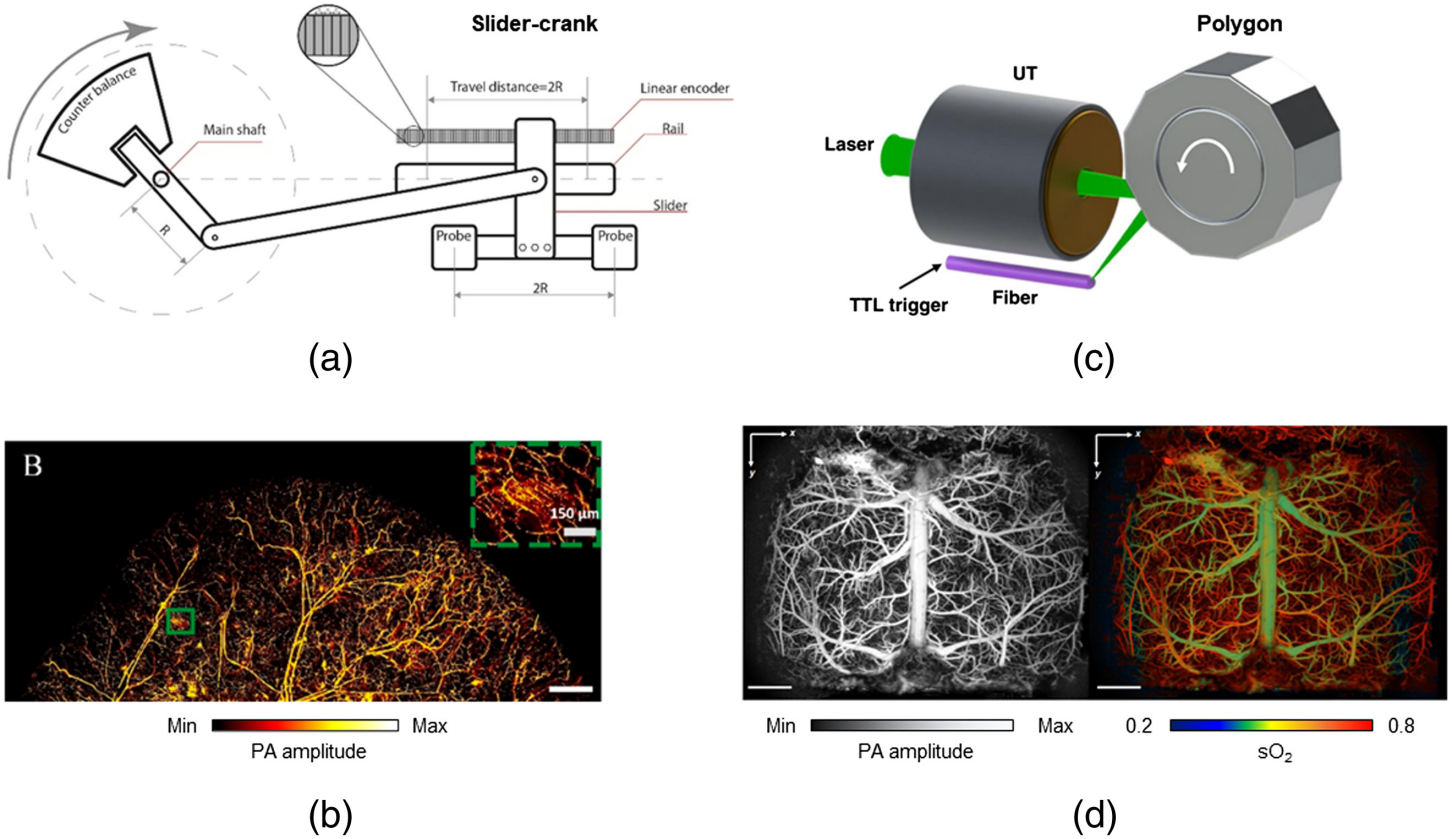
High-speed PAM system with mechanical scanning and optical scanning approaches. (a) Schematic of slider-crank scanner using two-channel PA probes. (b) High-resolution nude mouse ear PA image acquired by the slider-crank-scanner PAM system.^26^ (c) The illustration of high-speed PAM using a water-immersible polygon scanner with 12 facets. (d) PA images of the mouse brain vasculature and oxygenation over the entire cortex obtained from the polygon-scanner PAM system.^22^ Images (b) and (d) are reprinted from Refs. 26 and 22, respectively.

Mechanical scanning enables high-speed imaging with confocal and coaxial alignment of the light and sound over a large flat focal plane, and it consistently offers the best SNR. For example, the voice-coil scanner is an effective mechanical solution for high scanning speeds of up to 40 Hz B-scan rate.^25^ Nevertheless, as the scanning speed increases, the bulky voice-coil scanner head induces vibrations due to high driving forces, degrading its performance. Recently, a slider-crank scanner with a compact design and reduced vibration was reported.^27^ Using two sets of PA scanning probes, the slider-cranks scanner enabled a large scanning range of 24 mm^26^ [Figs. 3(a) and 3(b)]. However, it is still limited by the physical properties of the motor (e.g., speed and torque) and can provide a scanning speed of up to 32 Hz B-scan rate, which is much lower than most optical scanning systems.

### 3.2 Optical Scanning

Galvanometer scanners are known for fast and stable scanning in conventional optical microscopy. Galvanometer scanners in PAM were previously employed for steering the light beam in air, leading to the non-confocal alignment of the light and sound.^16^^,^^53^^,^^54^ Consequently, the SNR was relatively low and the optical scanning range was limited within the transducer’s detection zone. To address this issue, water-immersible optical scanners were used in PAM to concurrently and confocally steer both the optical beams and emitted acoustic waves. Although this approach increased the B-scan rate to several hundred Hz with a high SNR, the scanning range remains limited. For example, a semi-water-immersible galvanometer scanner achieved a B-scan rate of 500 Hz within a small FOV of ∼2.4  mm.^32^ On the other hand, employing cylindrically-focused or unfocused acoustic detection can expand the FOV significantly to ∼40  mm.^55^ However, this method has the cost of a reduced SNR.

Water-immersible MEMS scanners facilitated high-speed B-scan scanning at 400 Hz^29^^,^^30^ with a scanning range of 3 mm. However, these MEMS scanners had a highly reduced FOV unless operated at their resonant frequency. Furthermore, MEMS scanners were prone to thermal damage during prolonged use, resulting in distorted scanning patterns and warped PA images. Recently, Chen et al.^31^ introduced a torsion-bending-based MEMS scanner, achieving a B-scan rate of 400 Hz and a volumetric imaging speed of 1 Hz, covering a FOV measuring $1.5\times 2.5\;mm^{2}$. This system capitalized on the benefits of independent scanning axes, resulting in enhanced stability, particularly in aqueous environments. However, it was still limited by the relatively small FOV, compared with other mechanical or galvanometer scanners.

To achieve a large FOV, Zhu et al. used a 12-facet polygon-mirror scanner, which provided a B-scan rate of 2 kHz across an 11-mm scanning range^22^ [Figs. 3(c) and 3(d)]. Nonetheless, uneven step sizes across the scanning range degraded the PA image quality, necessitating data interpolation for final image reconstruction. Furthermore, steering the laser and ultrasound beams after the objective lens induced a curved scanning plane, further limiting the image quality.^56^ Moreover, the long travel distance for the ultrasound waves from the sample to the transducer increased PA signal attenuation.

## 4 Detecting systems

**Fig. 4:**
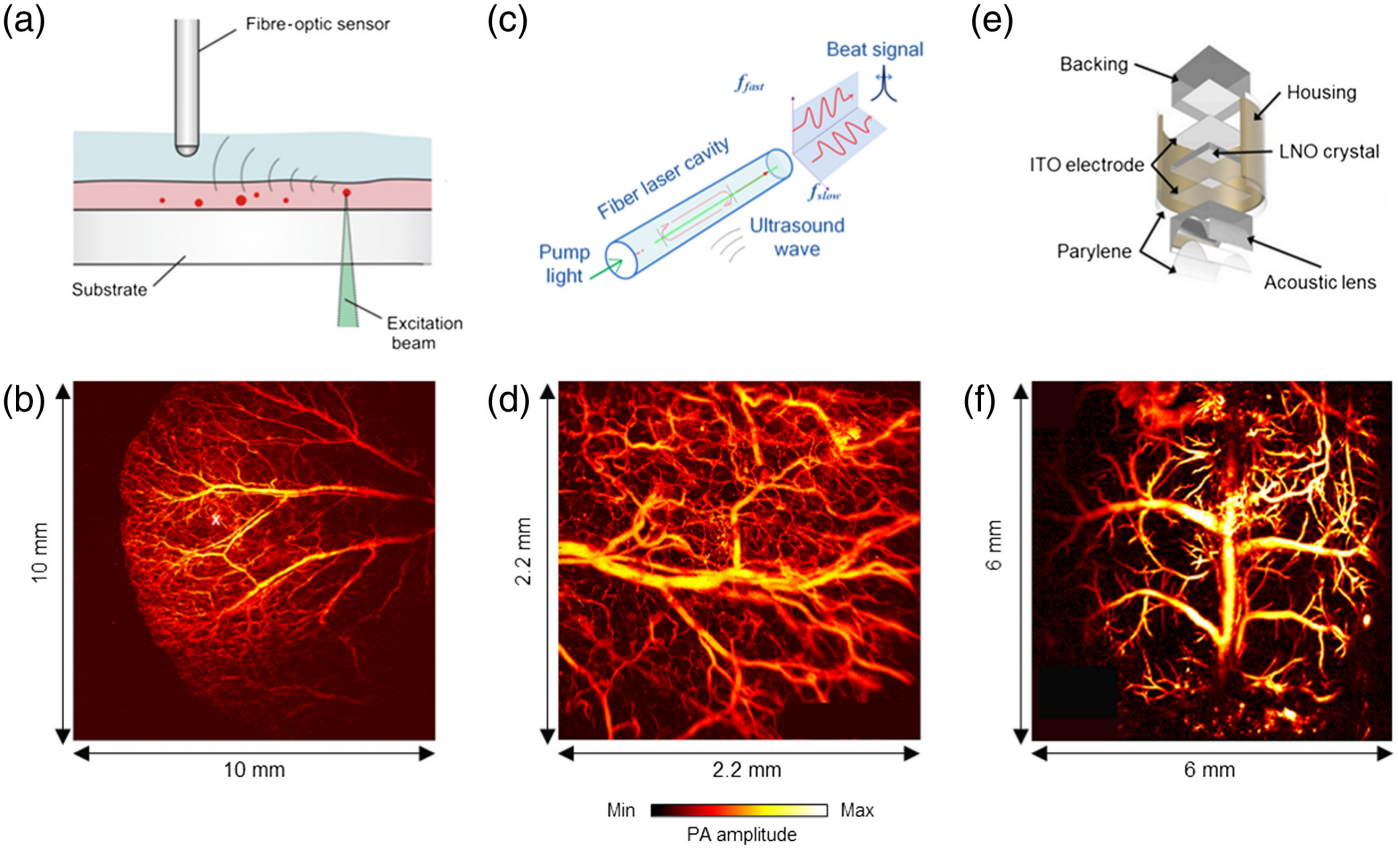
Various PA detection methods and their representative results by applying the optical scanning scheme. (a) Schematic of the planoconcave fiber-optic sensor.^16^ (b) *in vivo* PAM image of mouse ear obtained at 2 million A-lines per second. The scan area was 10  mm×10  mm, and the total acquisition time was 8 seconds. (c) Schematic of the fiber-laser based optic sensor method.^59^ (d) *In vivo* PAM image of the mouse ear at a C-scan frame rate of 0.2 Hz. The scanning FOV was $2.2\times 2.2\;{\mathrm{mm}}^{2}$. (e) Schematic of the transparent transducer method.^60^ (f) *In vivo* PAM image of whole cortical vasculature of the mouse brain. The total acquisition time was 5 s to scan an FOV of $6\times 6\;{\mathrm{mm}}^{2}$. Images (a), (b), and (c) are reproduced with permission from Refs. 16, 59, and 60, respectively.

Piezoelectric ultrasound transducers continue to dominate the PAM technologies due to their broad availability, high detection sensitivity, low cost, and ease of use. Nonetheless, piezoelectric transducers are often not transparent to light and thus complicate the PAM system design for light and sound alignment. Recently, optical ultrasound sensors have presented distinct advantages for PAM and gained increasing interest. Notably, the Fabry–Perot (FP) interferometer, functioning as an optical sensor, offers nearly isotropic spatial resolutions that are primarily determined by the size of the optical probing beam.^57^ The high-resolution imaging can be consistently maintained through 2D dense spatial sampling across the entire FOV. Traditionally, the imaging speed of the FP interferometer-based PAM system has been limited by the slow point-by-point raster scanning of the probing beam and the relatively low PRR of the PA excitation laser, typically operating at 50 Hz. However, a recent work conducted by the UCL group showcased a remarkable 32-fold increase in imaging speed.^58^ This improvement was achieved by simultaneously employing a total of eight parallel probing beams, all scanning over the sensor concurrently. For high-speed PA applications, a planoconcave fiber-optic sensor, which is a deposited polymer FP interferometer at the tip of an optical fiber, offers high detection sensitivity and an extensive bandwidth. Its wide acceptance angle (±90  deg) allowed for the fast detection of PA signals generated across a substantial FOV without requiring any sensor translation. In a recent work, Allen et al. adopted a custom-built fiber laser operating at a PRR of 2 MHz and demonstrated, for the first time, high-speed PAM using a planoconcave fiber-optic sensor [Figs. 4(a) and 4(b)].^16^ This high-speed PAM system employed a rapid galvanometer scanner to perform a raster scan of the laser beam across the sample surface while employing a stationary fiber-optic ultrasound detector for PA signal recording. Nonetheless, one limitation of the planoconcave fiber-optic sensor is its uneven sensitivity across a wide FOV, resulting in non-uniform PA image quality. Another approach used an unfocused side-looking fiber optic-sensor that was based on a dual-polarized fiber laser [Figs. 4(c) and 4(d)].^59^ This method combined stationary acoustic detection using the side-looking optical sensor with the fast laser beam scanning by a 2D galvanometer mirror. As a result, hemodynamic imaging was successfully demonstrated within a $2\times 2\;mm^{2}$ region at a volumetric frame rate of 2 Hz on the mouse ear. With a B-scan rate of 400 Hz, this technique allowed for imaging physiological dynamics in both trunk vessels and capillaries. Other optical ultrasound sensors, such as polymer micro-rings^61^ and Bragg-grating fibers,^62^ have recently been demonstrated as point-like detectors in PA imaging systems. These optical sensors often have sizes that are orders of magnitude smaller than their piezoelectric counterparts. However, a significant challenge faced by these optical sensors is the complexity of scaling up their fabrication while maintaining consistent optical properties such as the optical resonant wavelength, Q factor, and transmission efficiency. Therefore, they have not yet been applied on high-speed PAM systems. A comprehensive comparison of optical sensors and piezoelectric transducers can be found in the review article authored by Wissmeyer et al.^63^

Different from the conventional point-by-point scanning methods, parallel excitation and/or detection over a wide FOV allows for high-speed volumetric PA imaging. A recent study by Li et al. introduced a high-speed PAM implementation known as PA Topography through an Ergodic Relay (PATER).^64^ This method was based on the simultaneous encoding of PA signals from a wide FOV based on their distinct time-delay characteristics. In PATER, each individual excitation laser pulse enabled the parallel detection of encoded PA signals using a single-element ultrasound transducer. These signals were subsequently mathematically decoded to reconstruct a 2D projection image. By incorporating a point-by-point scanning calibration process, PATER showcased a topographic frame rate of 2 kHz over a FOV of $8\times 6\;{\mathrm{mm}}^{2}$. This approach was applied to visualize the blood pulse wave velocity and monitor the circulation of melanoma cells within the mouse brain. PATER’s imaging speed was primarily limited by the acoustic transit time within the ergodic relay as it eliminated the need for optical or acoustic beam scanning. However, the existing calibration method lacks depth information; thus, it can only provide topographic images. Xia et al. introduced multifocal PA-computed microscopy, utilizing a 2D microlens array and a 512-element full-ring ultrasonic transducer array.^65^ They transmitted 1800 optical foci within the transducer’s focal plane and performed raster scanning with a 25  μm step size. As a result, they obtained a cross-sectional PAM image across a FOV of $10\times 10\;mm^{2}$ in just 36 s. However, the resolution (∼36  μm) was quite poor in comparison with other PAM systems. This approach was also applied in ultraviolet PAM by Imai et al., who developed a high-throughput multifocal Ultraviolet PAM (MF-UV-PAM) that employed a linear microlens array, which requires only 1D scanning for volumetric imaging by utilizing a 256-channel data acquisition system.^66^ They accomplished a lateral resolution of ∼1.6  μm and achieved a remarkable 40-fold increase in imaging speed compared with conventional raster scanning. The transparent ultrasound transducer is another novel approach aimed at simplifying and accelerating PAM systems.^67^^,^^68^ As shown in Figs. 4(e) and 4(f), with this design, the laser beam can pass through the optical transparent transducer, without the need for an optical acoustic combiner or/and steering mirror. In the context of high-speed PAM, a cylindrically-focused transparent high-frequency ultrasound transducer (CFT-UT) combined with a galvo scanner was used.^60^ This setup managed to achieve a B-scan frame rate of 500 Hz over a scanning range of 9 mm. However, it is important to note that the current prototype of the CFT-UT exhibited non-uniform optical transparency and detection sensitivity, resulting in non-uniform PA images (Table 1).

**Table 1:** Specifications of PAM with high imaging speed. PRR, pulse repetition rate; $f_{c}$, center frequency of ultrasound sensor; Δf, bandwidth of ultrasound sensor; FOV, field-of-view; δx, lateral resolution; δz, axial resolution; PT, piezoelectric transducer; and FP, Fabry–Pérot.

References	Wavelength	PRR	E	Scanning/image formation method	Detection method	$f_{c}$	Δf	B-scan rate	C-scan rate	FOV	δx	δz
24	532 nm	600 kHz	150 nJ	2D galvanometer	PT	10 MHz	—	400 Hz	2 Hz	$1\times 1\;{\mathrm{mm}}^{2}$	6 μm	—
15	532 nm	500 kHz	1 μJ	1D MEMS scanner	PT	50 MHz	100%	400 Hz	1 Hz	$3\times 2\;{\mathrm{mm}}^{2}$	3 μm	15 μm
16	532 nm	2 MHz	500 nJ	2D galvanometer	FP fiber sensor	—	60 MHz	500 Hz	1 Hz	$5\times 5\;{\mathrm{mm}}^{2}$	—	—
23	532, 545, and 558 nm	160 kHz	150 nJ	2D galvanometer	PT	10 MHz	—	—	—	—	7 to 8 μm	—
19	532 to 695 nm (10-wavelengths)	300 kHz	116 to 756 nJ	2D galvanometer	PT	20 MHz	42.2%	480 Hz	0.98 Hz	$3\times 3\;{\mathrm{mm}}^{2}$	—	—
17	528 and 558 nm	500 kHz	80 nJ	2D galvanometer	PT	50 MHz	76%	—	—	$4.5\times 3.5\;{\mathrm{mm}}^{2}$	7.8 μm	41 μm
20	532 and 558 nm	1 MHz	80 nJ	1D polygon scanner	PT	50 MHz	78%	477.5 Hz	1 Hz	$12\times 5\;{\mathrm{mm}}^{2}$	6.3 to 21 μm	36 μm
21	532 and 558 nm	1 MHz	150 nJ	1D MEMS scanner	PT	40 MHz	110%	1 kHz	6 Hz	0.75 mm	2.7 μm	30 μm
25	570 nm	4 kHz	100 nJ	Voice-coil scanner	PT	75 MHz	133%	40 Hz	—	1 mm	3.4 μm	15 μm
27	532 nm	25 kHz	200 nJ	Slider-crank scanner	PT	25 MHz	74.55%	30 Hz	—	10 mm	10.2 μm	76 μm
26	532 nm	200 kHz	—	Slider-crank scanner	PT	50 MHz	—	32 Hz	—	24 mm	3.4 μm	37 μm
32	532 nm	500 kHz	200 nJ	1D galvanometer	PT	50 MHz	82%	500 Hz	—	2.4 mm	7.5 μm	33 μm
29	532 nm	100 kHz	100 nJ	1D MEMS scanner	PT	50 MHz	100%	400 Hz	0.8 Hz	3 mm	2.4 μm	26 μm
30	532 nm	10 kHz	—	2D MEMS scanner	PT	50 MHz	100%	50 Hz	0.25 Hz	$9\times 4\;{\mathrm{mm}}^{2}$	3.6 μm	27.7 μm
31	532 and 558 nm	200 kHz	180 nJ	2D MEMS scanner	PT	50 MHz	100%	400 Hz	1 Hz	$1.5\times 2.5\;{\mathrm{mm}}^{2}$	3.8 μm	34 μm
22	532 and 558 nm	800 kHz	200 nJ	1D Polygon scanner	PT	40 MHz	100%	2 kHz	0.3 Hz	$8\times 7.5\;{\mathrm{mm}}^{2}$	10 μm	33 μm
58	720 nm and 850 nm	200 Hz	—	2D galvanometer	FP sensor array	—	—	17 Hz	0.1 Hz	$10\times 10\;{\mathrm{mm}}^{2}$	—	—
59	532 nm	160 kHz	300 nJ	2D galvanometer	Fiber laser sensor	22 MHz	70%	400 Hz	2 Hz	$2\times 2\;{\mathrm{mm}}^{2}$	3.2 μm	110 μm
64	532 nm	2 kHz	—	PATER	PT	20 MHz	56%	2 kHz	—	$8\times 6\;{\mathrm{mm}}^{2}$	110 μm	—
65	532 nm	10 Hz	12 mJ	2D microlens array	Ring transducer array	5 MHz	80%	—	36 sec	$10\times 10\;{\mathrm{mm}}^{2}$	29.4 μm	—
66	266 nm	10 kHz	—	1D microlens array with multifocal excitation	1D array PT	50 MHz	—	—	16 min	$7\times 10\;{\mathrm{mm}}^{2}$	1.6 μm	40.9 μm
60	532 nm	800 kHz	1 μJ	1D galvanometer	Cylindrically focused transparent PT	34 MHz	25.8%	500 Hz	10 s	9 mm	10 μm	208 μm

Often in PAM, a volumetric image can be generated by 2D raster scanning with depth-resolved PA signals, and thus depth scanning is not necessary. With that said, due to the limited depth of focus of the light beam in OR-PAM, depth scanning is sometimes needed to extend the imaging depth on thick samples. Several recent works focus on extending the depth of focus of the light beam and thus increasing the volumetric imaging speed. Techniques such as using dual non-diffracting Bessel beams,^69^ synthetic Bessel light needles,^70^ and deep learning methods^71^ have been employed to extend the depth of field in OR-PAM. These advancements enable an extended focus and faster volume scans. Bessel beams, in particular, have shown promise in maintaining a narrow focal region over a longer range,^70^ which is beneficial for high-resolution imaging over extended depths.

## 5 Computational Techniques

As the imaging speed of PAM continuously increases, the inherent drawbacks with a high imaging speed may not be addressed by improving the system configurations alone. Such drawbacks include the spatial undersampling, scanning-line misalignment, reduced SNR, and deteriorated spatial resolution. Advanced computational techniques can be used to enhance the high-speed PAM image quality, mostly by focusing on the post-imaging processing.

### 5.1 Upsampling Methods

#### 5.1.1 Spatial undersampling

One of the primary ways computational techniques have been used to speed up PAM imaging is to reduce the number of spatial sampling events required for a complete PAM scan, particularly when the laser’s PRR is limited. Although often less effective than modern deep learning techniques, non-deep learning approaches can be more interpretable and, without the need for extensive training datasets or complex data augmentation regimes, can still be more generally robust. Similar to compressed sensing, these non-deep learning approaches can recover sparsely sampled data by exploiting known information about the data’s underlying sparsity to overcome typical Nyquist–Shannon limitations. One method utilized to transform sparsely sampled PAM data into an approximation of the fully sampled images frames the recovery task as a matrix completion problem. In Lui et al.’s work,^72^ the alternating direction method of multipliers (ADMM) based low-rank and sparse matrix recovery method is used on sparse PAM data to enable fast vascular imaging. By enforcing a sparsity constraint (total variation norm) and a low-rank constraint (nuclear norm), this optimization problem can be solved with ADMM, resulting in superior signal recovery compared with other contemporary algorithms such as GoDec.^73^ Dictionary learning is another popular non-deep learning approach for recovering quickly acquired, sparsely sampled PAM images. In dictionary learning, a sparsely coded dictionary is used to represent the data, whereby the dictionary is iteratively updated to minimize the representation error while maintaining a certain sparsity constraint. The desired result is a dictionary representation that best represents the acquired data given the specified level of sparsity. In Sathyanarayana et al.’s work,^74^ a dictionary learning technique known as K-SVD is implemented with a dictionary size of 1024, sparsity of 20, 5 inner loop iterations of weighted rank 1 SVD approximation, and 5 outer loop iterations of K-SVD alternating minimization. This method was able to recover randomly downsampled PAM images of 50% and 75% sparsity with a minimal impact on the peak-signal-to-noise ratio (PSNR) compared with the fully sampled images. This work was later expanded upon in another work by Sathyanarayana et al.,^75^ which could recover hemodynamic parameters from undersampled multi-parametric PAM data with up to eight times undersampling, with less error than bicubic interpolation. More recently in Sulistyawan et al.’s work,^76^ a curvelet transform was used to reconstruct the boundary and separability of cells in images randomly undersampled up to eight-fold with better noise rejection and edge recovery compared with nearest neighbor interpolation with a smoothing filter.

**Fig. 5:**
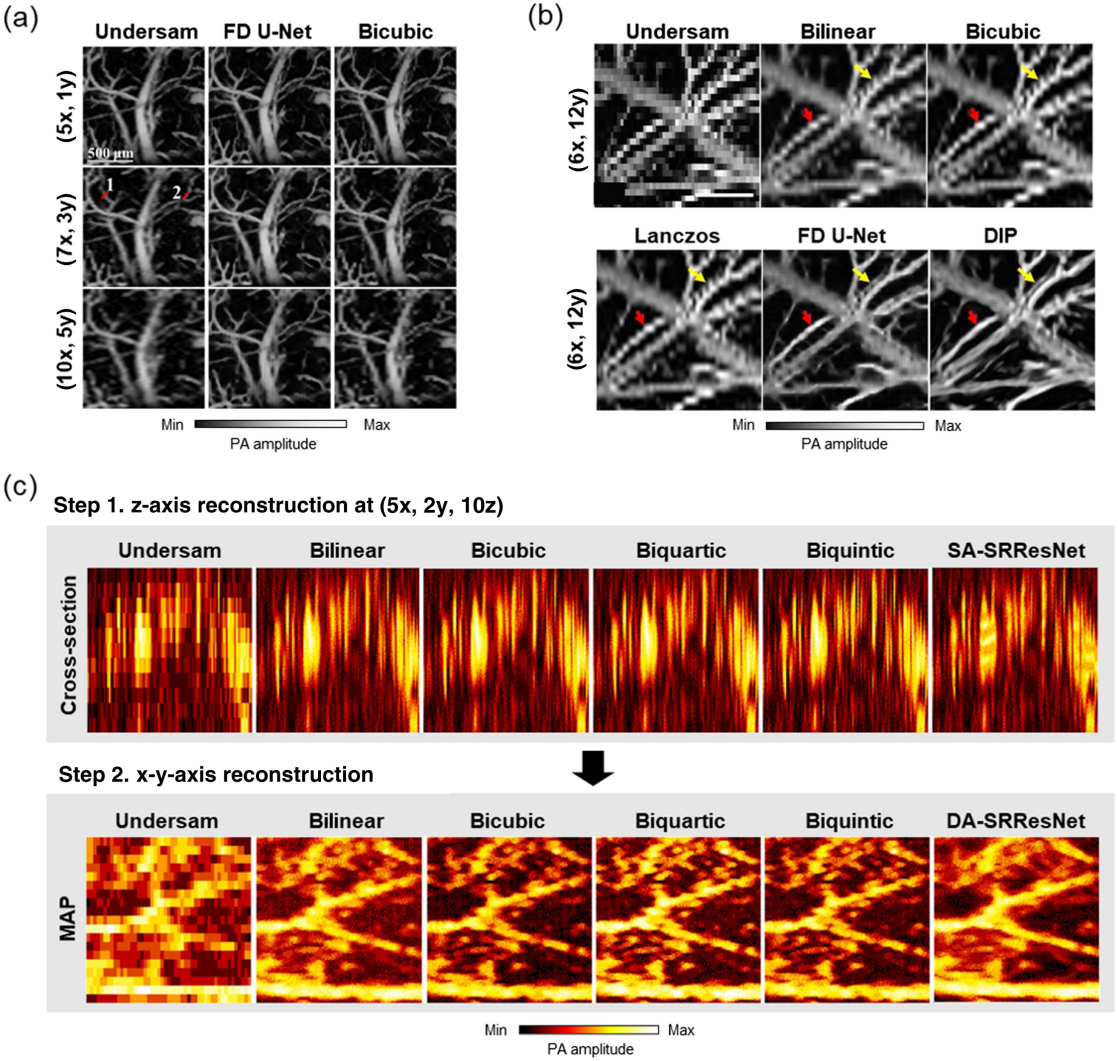
Deep learning methods for upsampling PAM data. (a) PAM MAPs upsampled using FD U-Net with supervised training at various downsampling ratios (i.e., ratios [Dx, Dy]) compared with interpolation.^34^ (b) PAM MAPs upsampled using the unsupervised DIP iterative method compared with interpolation methods and FD U-Net.^35^ (c) PAM data upsampled in 3D using a combination of axial z-axis upsampling (SA-SRResNet) and lateral x-y-axis upsampling (DA-SRResNet).^77^ Images (a), (b), and (c) are reprinted with permission from Refs. 34, 35, and 77, respectively.

DiSpirito et al. published the first work to use deep learning to accelerate PAM through reconstructing undersampled images.^34^ In this work, several deep learning model architectures were compared, and a modified fully dense U-Net (FD U-Net) was found to perform the best at the various downsampling ratios. At each of these downsampling ratios, FD U-Net was able to reconstruct vessel structures with smoother edges and less jagged aliasing artifacts than bicubic interpolation as the model learned vasculature features during its supervised training [Fig. 5(a)]. This sense of vesselness was reinforced using a multifactorial loss function that included pixelwise loss in the spatial and Fourier domains, as well as perceptual loss in the form of structural similarity index measure and PSNR.

Soon after, Zhou et al.^78^ reported that a convolutional neural network architecture with 16 residual blocks and 8 squeeze-and-excitation (SE) blocks was able to reconstruct undersampled PAM images. Unlike DiSpirito et al.,^34^ the proposed model was trained on experimentally downsampled PAM images of 25% and 6.25% effective pixels respectively, rather than synthetically downsampled images. The trained model was shown to outperform bicubic interpolation and several other popular single image super-resolution deep learning architectures. The authors emphasized that the high quality of the images reconstructed using their technique was in part due to training on experimentally downsampled data with a VGG19 perceptual loss function.

Rather than using a traditional supervised deep learning approach, whereby one needs a large dataset of ground truth high-resolution PAM images, Vu et al.^35^ used an unsupervised method to upsample PAM images [Fig. 5(b)]. In this work, the deep image prior (DIP) technique utilized a modified FD U-Net to iteratively convert Gaussian noise into a given undersampled PAM image using the known downsampling binary mask. Though this unsupervised method had a much longer inference time due to the need to iteratively retrain for every new image, it also benefited from being able to create more natural vessel structures, without the need for ground truth or the presence of transposed convolution checkboard artifacts.

Most spatial undersampling works in PAM have been restricted to working with 2D maximum amplitude projection (MAP) images and only with lateral (or x-y) direction undersampling. However, PAM images are originally acquired as 3D volumes, with a depth direction z in addition to the usual x-y directions shown in MAP images. To utilize a lower sampling frequency and reduce PAM’s volumetric data size, Seong et al.^77^ used a modified super-resolution ResNet (SRResNet) to improve PAM data undersampled in 3D volume (along the z and x-y directions) [Fig. 5(c)]. This work used two deep learning models, a modified single axis SRResNet and a dual axis (DA) SRResnet, to perform upsampling along the z-direction and the x-y directions respectively. Similar to DiSpirito et al.’s work, Seong et al. used fully sampled data during model training as ground truth and then simulated undersampled data by downsampling along the x-, y-, and z-directions respectively, with the ability to vary the downsampling ratio used for each spatial dimension. The model also performed well on experimentally downsampled test data.

#### 5.1.2 Temporal undersampling

In addition to reducing the required spatial sampling density of PAM, deep learning has also been used to reduce the number of temporal frames required to form PA localization microscopy images. In PA localization microscopy, N number of frames capturing localized targets are combined to attain a high-quality dense localization-based image. This image formation technique can acquire high resolution images of vasculature at the expense of poor temporal resolution as a sufficient number of frames must be acquired and combined to form a high-quality image. To circumvent the speed limitation, Kim et al.^36^ used a deep learning model based on the Pix2Pix generative adversarial network (GAN) framework to convert low quality (low N number) images into their higher quality (high N number) equivalents. A 3D U-Net was used as the generator, with seven 3D convolutional layers and roughly 43 million trainable parameters, and the discriminator consisted of five convolutional layers connected in series and contained ∼5 million trainable parameters. Model performance was exhibited after being trained on various input N frame total values, with potential imaging speed enhancements of ∼12-fold.

### 5.2 Computational Correction Methods

#### 5.2.1 Alignment of the scanning system

**Fig. 6:**
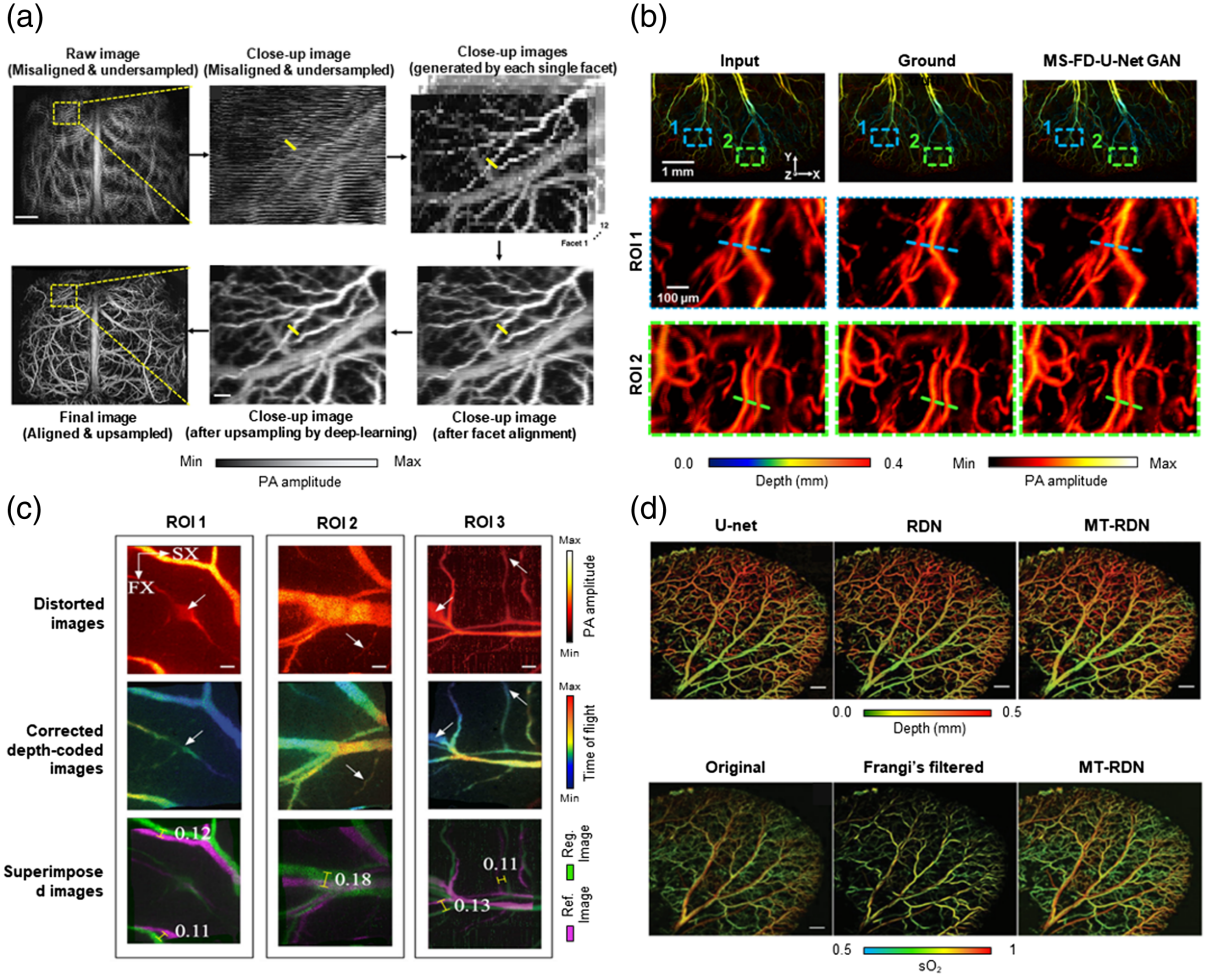
Improving PAM images by (a) aligning polygon facets in a fast polygon scanning system combined with deep learning FD U-Net upsampling,^22^ (b) recovering misaligned signal from bidirectional scan using deep learning,^37^ (c) correcting MEMS scanning distortion using a deep learning spatial weight matrix (SWM) with a dimensionality reduction,^38^ and (d) improving the image quality of low energy PAM using MT-RDN.^39^ Images (a) and (b)–(d) are reprinted with permission from Refs. 22 and 37–39, respectively.

Computational techniques have also been used alongside fast hardware to help speed up PAM through performing imaging system corrections. For example, the manufacturing of polygon scanning mirrors can introduce minor imperfections that present as distortions and misalignments among the various polygon facet faces. In Zhu et al.’s work,^22^ a combination of affine transformations between facet images and deep learning upsampling was used to correct for this facet misalignment [Fig. 6(a)]. The calibrated affine transformations removed most of the global distortions that were caused by polygon facet misalignment, and the remaining minor local distortions were mitigated further by the deep learning upsampling procedure.

When using high-speed water-immersible MEMS scanners, the consecutively acquired bidirectional PAM images are typically misaligned because of unstable mirror performance. This usually results in one of the bidirectional scans being discarded, thereby doubling the scanning time necessary to meet Nyquist requirements. In Kim et al.’s work,^37^ the misalignment in these bidirectional scans was corrected using a deep learning network based on a FD U-Net, known as multiscale (MS) FD U-Net, in a GAN configuration [Fig. 6(b)]. When compared with traditional enhancement filtering (such as bicubic, bilateral, and median filters) and previously reported deep learning networks (Dense GAN and FD U-Net), the proposed MS FD-U-Net was shown to be superior in terms of perceptual similarity to the ground truth.

MEMS scanning mirrors are often used to accelerate OR-PAM. However, the nonlinear tilt angular-voltage characteristic of a MEMS mirror introduces distortions into the MAP image. In addition, other factors such as the size of the airy disk, ultrasonic sensor properties, and thermal effects can decrease the resolution. Ma et al.^38^ proposed a spatial weight matrix (SWM) with a dimensionality reduction for image reconstruction that aimed to improve these downsides of traditional MEMS scanners [Fig. 6(c)]. The three-layer SWM was calibrated to the constant distortion factors of the system, allowing for both spatial dependent distortion correction and 3D deconvolution. An ordinal-valued Markov random field and Harris Stephen algorithm were used in tandem with a modified delay-and-sum method during reconstruction. Using this multistep adaptive calibration approach, Ma et al. showcased that many of the distortions caused by MEMS system characteristics could be effectively mitigated.

#### 5.2.2 Denoising of low laser pulse energy

Clinical translation of high-speed OR-PAM requires safe laser energy levels as regulated by the American National Standards Institute (ANSI) safety standards. This necessitates a tradeoff between laser energy, imaging speed, and image quality. Conventionally, one may sacrifice laser pulse energy and thus image quality to maximize imaging speed. The work of Zhao et al.^39^ shows that deep learning can circumvent this tradeoff by improving the image quality caused by low laser energy under the ANSI limitations [Fig. 6(d)]. By integrating multi-supervised learning, dual-channel sample collection, and a reasonable weight distribution, the proposed deep learning model, known as multitask residual dense network (MT-RDN), was shown to have superior image quality, even with a 16 to 32-fold reduction in laser energy. With three subnetworks, the proposed model learned the multi-supervised task of simultaneous image denoising, super-resolution, and vascular enhancement. Zhao et al. also demonstrated the robustness of their technique with its ability to extrapolate to ear vasculature after only being trained on brain vasculature images.

## 6 Conclusion and Outlook

Imaging speed is an essential priority of PAM when monitoring biological functions and capturing dynamic changes in biological processes. Due to the fast development of key technologies, including (1) light sources, (2) scanning systems, (3) detecting systems, and (4) computational techniques, PAM has experienced remarkable advances in terms of imaging speed in the last decade.

For the light source, the laser’s PRR plays a key role in determining the A-scan rate. Therefore, increasing the maximum PRR is an ideal approach for high-speed imaging. In PAM, even an MHz-level PRR is allowable because the target is located at a shallow depth. However, conventional PAM systems used slow bulky solid-state lasers utilizing the crystal as the gain medium, which was vulnerable to thermal lensing effect and thermal damage, thus limiting the input threshold power for a high PRR. Therefore, the emergence of fiber lasers has become a powerful driving force for ultrafast PAM. Because optical fibers have strong thermal resistance and are less affected by the thermal lensing effect, even commercial fiber lasers can produce the pulse energy of microjoules at an MHz-level PRR. Harnessing the development of commercial single-wavelength fiber lasers, techniques using nonlinear optical effects (e.g., SRS) have been utilized for multi-wavelength PA excitation, especially targeting oxy- and deoxy-hemoglobin. Consequentially, PAM using an MHz-level SRS source has enabled the monitoring of rapid ${\mathrm{sO}}_{2}$ changes, observing the initial dip from cerebral micro-vessels without repeated simulation, and achieving rapid whole-brain hemodynamics imaging. However, current light sources still have challenges for ultrafast PAM, such as the limited wavelength tunability and the low pulse energy. For example, when coupled at an MHz-level PRR, the optical fiber is prone to thermal damage, which prevents a stable output at tunable wavelengths. Further optimizing high-speed tunable lasers may focus on improving the fiber characteristics such as the effective area, nonlinear efficiency, and thermal resistance.

The development of scanning systems has proven to be an effective approach to optimizing the scanning speed over a large FOV. Mechanical scanning systems excel in high SNRs but often have low speed. Traditional optical scanning systems have high scanning speed while maintaining a good SNR, although they often have limitations in the FOV. Recent advancement of optical scanning systems has pushed the PAM speed limit to approximately the physical limit determined by ultrasound propagation. Various methods to detect the PA signals have been suggested as an alternative approach. One solution might be remote sensing technology that detects the local optical reflection change induced by an acoustic pressure rise.^79^^–^^81^ The PA remote sensing does not rely on acoustic wave propagation and thus can achieve a higher signal detection speed. Optical sensors have emerged as a promising solution for achieving faster signal detection speeds, mostly coupled with fast optical scanning of the PA excitation beam. They offer several advantages, such as a compact size, wide reception angle, broad detection bandwidth, strong responsiveness in the low-frequency range, and seamless integration with the PA light path. However, optical sensors face challenges when it comes to multiplexing as even minor fabrication inaccuracies can significantly affect their performance. Another drawback is their susceptibility to environmental instability in biological settings. For example, micro-ring resonators can be sensitive to surface contamination, causing scattering and absorption losses, and FP interferometers can be affected by temperature variations, altering the properties of the polymer spacer. This instability in biological environments can lead to a rapid decline in sensor sensitivity and hinder their use in longitudinal *in vivo* studies.

With the introduction of ever more efficient computing resources over the past few years, there has been an explosion of new computational techniques to address the issues of spatial and temporal undersampling, as well as misalignment, distortion, and low laser energy correction. Hardware limitations, such as the limited laser PRR, have been transcended by upsampling the spatially/temporally undersampled PAM data, and already-fast hardware can overcome traditional sampling-imposed limitations by decreasing the sampling density required to achieve high image quality. Additionally, computational techniques have facilitated faster scanning mechanisms (e.g., polygon or MEMS scanners) by correcting the misalignment or distortion induced by rapid scanning speeds. Similarly, these techniques have enabled high-speed PAM while using laser pulse energy under the ANSI limit. Despite these many advances in the computational enhancement of PAM, there still exist significant hurdles regarding the need for large ground truth datasets. These datasets are often time consuming to gather, and ground truth can sometimes be difficult to access, especially in *in vivo* settings. Simulation or other synthetic data generation approaches have been used to address this issue. However, without appropriate augmentation and regularization techniques, there can exist a domain gap when applying deep learning models trained on synthetic datasets to improve experimentally acquired, complex *in vivo* images. Some steps have been taken to address this limitation by creating a large high-resolution open-source dataset,^82^ using experimentally-acquired ground-truth images,^37^^,^^78^ or adopting the realm of less data-hungry self-supervised or unsupervised deep learning methods.^35^ However, future work is still needed, likely by incorporating multiple approaches to address system specific-requirements with a fusion of hardware innovations and tailored computational interventions, to speed up PAM systems in a less data-hungry and more interpretable/experimentally-grounded way. It should be noted that the new computational, data-driven techniques also pose their own inevitable risks and challenges. One set of difficulties when using deep learning emerges from the nature of these “black-box” models as being fundamentally challenging to interpret.^83^^–^^85^ This issue of interpretability is then exacerbated by the occasional pattern of deep learning models to add features not based in reality, so called “hallucinations,” when confronted with uncertain inputs.^86^ For example, when faced with corrupted input, such as aliased or noisy PAM data, it is possible for deep learning models to add vessels that do not exist in areas of model uncertainty. Finally, without the use of standardized, open-source, deep learning datasets and the release of code as open source, it can be difficult to build upon the work of other researchers and quantify method improvements.^87^ Many of these issues are still being addressed by deep learning practitioners, such as with more realistic simulation techniques, data augmentation, regularization, and model uncertainty quantification; thus this remain an area of growth for the field.

Overall, we expect that more efforts for implementing high-speed PAM will continue for monitoring the rapid dynamic changes in biomedical processes, enabled by further development of key technologies. Recognizing the current limitations, breaking through the drawbacks mentioned above, and adopting the optimal combination of each technology will lead to the realization of ultimate high-speed PAM for both fundamental research and clinical translation.

## Data Availability

Data sharing is not applicable to this article as no new data were created or analyzed.
